# Supplementary material for: Impact of COVID-19 Pandemic on Thyroid Surgery in a University Hospital in South Korea
Source: Cancers (Basel). 2022 Sep 5;14(17):4338. doi: 10.3390/cancers14174338 (PMC9454546; doi:10.3390/cancers14174338)
Supplement: Supplementary file 1 [file cancers-14-04338-s001.zip › cancers-1845983-supplementary.pdf]

Supplementary Table S1. Numbers of outpatients in author's institution and COVID-19 patients in Korea.

Year	Month	New patients	Follow up patients	Total outpatients	Numbers of COVID-19 patients in Korea	Correlation*	p-value
2019	1	109	388	497	0	NA	NA
	2	106	327	433	0		
	3	104	468	572	0		
	4	87	472	559	0		
	5	121	461	582	0		
	6	108	442	550	0		
	7	121	511	632	0		
	8	107	565	672	0		
	9	100	442	542	0		
	10	107	520	627	0		
	11	114	504	618	0		
	12	119	484	603	0		
2020	1	105	563	668	11	-0.17	0.598
	2	80	477	557	2920		
	3	75	358	433	6855		
	4	55	315	370	979		
	5	88	322	410	703		
	6	60	374	434	1331		
	7	84	438	522	1506		
	8	77	361	438	5641		
	9	58	309	367	3865		
	10	93	353	446	2700		
	11	92	371	463	7688		
	12	72	368	440	26527		

2021	1	62	329	391	17471	0.668	0.018
	2	75	324	399	11467		
	3	75	467	542	13415		
	4	101	386	487	18927		
	5	76	359	435	18331		
	6	107	425	532	16623		
	7	81	359	440	41374		
	8	87	416	503	53007		
	9	87	394	481	59859		
	10	109	393	502	53421		
	11	100	415	515	82527		
	12	138	472	610	183604		

Reference: Center Disease Control Headquarters in Korea, http://ncov.mohw.go.kr/index.jsp

* Pearson's product-moment correlation between total outpatients and numbers of COVID-19 patients
